# Providing information for decision-making in the Nigerian Polio Eradication Program, 2016-2020

**DOI:** 10.11604/pamj.supp.2023.45.2.39489

**Published:** 2023-09-07

**Authors:** Tesfaye Erbeto, Philip Bammeke, Aron Aregay, Zabihullah Kamran, Ahmed Ibrahim, Nnamdi Usifoh, Usman Adamu, Bolu Omotayo, Fiona Braka, Eunice Damisa, Walter Kazadi, Faisal Shuaib

**Affiliations:** 1 World Health Organization Country Office, Abuja, Nigeria,; 2 Centers for Disease Control and Prevention, Atlanta, Georgia, United States,; 3 United Nations Children’s Fund Nigeria Country Office, Copenhagen, Denmark,; 4 African Field Epidemiology Network, Abuja, Nigeria,; 5 National Primary Healthcare Development Agency, Abuja, Nigeria,; 6 National Emergency Operation Centre, Abuja, Nigeria

**Keywords:** Vaccination campaign, dashboard, polio, local government areas, monitoring, surveillance, information, decision-making

## Abstract

Nigeria made a coordinated effort to be certified by the World Health Organization’s African Region for interrupting endemic transmission of wild poliovirus type-1 (WPV1) in August 2020 as a response to the resurgence of WPV1 cases in August 2016 after going two years without a case. The NEOC Data Working Group (DWG) was instrumental in providing quality and timely surveillance and campaign information for decision-making in order to interrupt WPV1 transmission and provide data toward documentation of its elimination for regional certification. The polio pre-campaign dashboard was used to assess the level of preparedness for Oral Poliovirus Vaccine (OPV) polio supplementary immunization activities (SIA) at three weeks, two weeks, one week, and three days to the start of each campaign implemented during 2016-2020. The administrative tally sheet, independent monitoring survey, and Lot Quality Assurance Sampling (LQAS) survey data collected and shared from the implementation level were analyzed by the EOC DWG to provide information by person, place, and time. Using a 90% threshold in LQAS surveys defining quality SIAs, the proportion of Local Government Areas (LGAs) in Nigeria’s states in which post-SIA LQAS surveys were conducted that met this threshold were assessed over time. The highest level of preparedness attained by 3 days to a polio campaign during August 2016-February 2020 was 95% and the lowest attained was 77%. The admin, independent monitoring, and LQAS data analysis results were given to EOC working groups for assessing the performance and quality of each campaign. Twenty-twenty five percent of LGAs that failed LQAS were identified for repeat vaccination. Further, acute flaccid paralysis and environmental surveillance data and laboratory results were analyzed and shared with NEOC and partners. The government and partners used the information generated by the Data Working Group to take evidence-based action including determining the scope of the polio campaign, intensification of surveillance and routine immunization activities, and special intervention activities. On average, 12% of the 774 LGAs were identified as polio high risk LGAs for intervention using selected surveillance, routine immunization (RI), SIAs, and other relevant data sets. National Emergency Operation Centre Data Working Group provided quality and timely information that supported decision-making processes for the polio program in Nigeria. The quality and timely information enabled the NEOC to make evidence-based and timely decisions that contributed to gap identification and decision-making.

## Introduction

In 2016, Nigeria was the last remaining country of the World Health Organization (WHO) Region of Africa to confirm transmission of wild poliovirus type 1 (WPV1). In August 2020, based on detailed surveillance and immunization data from Nigeria, the African Regional Certification Commission certified the Region as free of indigenous WPV transmission [1]. Since 2012, the National Emergency Operation Centre (NEOC) in Nigeria has been responsible for providing leadership, coordination, and oversight of planning, supervision, and implementation of all polio eradication activities [2] and is comprised of six Working Groups: Strategy, Operations, Communications, Surveillance, Nomadic Populations, and Data. Before NEOC’s establishment, the management of polio eradication program-related data by the government and partner agencies in Nigeria was fragmented and often redundant [3]. The Data Working Group (DWG) centralized data management. Datasets include AFP surveillance and investigations, supplementary immunization activities (SIAs) implemented as national immunization days (NIDS) or sub-nationally in high-risk states, special interventions (e.g. vaccination in security-compromised areas), and all other immunization-related datasets. The DWG provides complete, timely, and quality information for planning, decision-making, and intervention [4]. The DWG is comprised of data experts from the government and four in-country partners: the African Field Epidemiology Network (AFENET), the Bill and Melinda Gates Foundation (which funded the establishment of the EOCs), UNICEF, and WHO [5]. AFENET is an implementing partner of the US Centers for Disease Control and Prevention (CDC); CDC country office staff are also NEOC members.

Nigeria has 36 states plus the Federal Capital Territory (FCT) comprised of 774 Local Government Areas (LGAs), further subdivided into wards. The NEOC provides guidance, protocols, and policy directives for the implementation of all eradication activities for state-level EOCs in seven historically high-risk states (Bauchi, Borno, Kaduna, Kano, Katsina, Sokoto, and Yobe) and immunization staff in other states. The NEOC provided valuable support during the 2014 Ebola virus disease outbreak, multiple measles outbreaks, and the ongoing COVID-19 pandemic response [6-8]. This report describes the DWG process and products, and specifically how they pertain to NEOC activities for the polio eradication efforts in Nigeria.

## Methods

**Data collection:** oral poliovirus vaccine campaign data are collected from vaccination teams and independent monitors and shared with the next level before, during, and after the campaigns. These data are collated at progressive levels (ward, LGA, and state) and shared with each of the seven WHO Zonal Offices. These offices in turn merge the data sets, run data quality checks seek corrections, and submit data to the state EOCs and NEOC. Timelines for data submission are provided for each level. At the national level, the DWG performs further data quality checks in place for the various data instruments and in addition through the national Monitoring and Accountability (M&A) officers at the NEOC that function as liaisons for the NEOC in contacting the 36 states and FCT on all polio-related activities, and seeks corrective feedback, before the DWG analyzes data to produce a comprehensive report.

**Oral poliovirus vaccine supplementary immunization activities**

**Figure 1:**
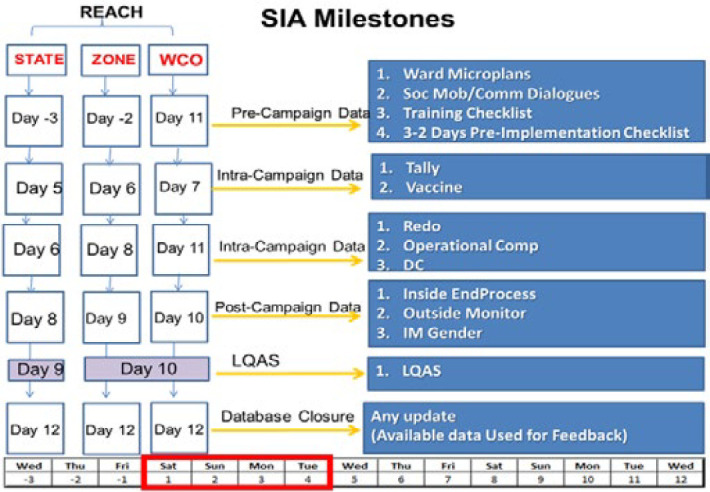
oral poliovirus vaccine supplementary immunization activity campaign data flow chart, Nigeria, 2012-2020

**Before supplementary immunization activities:** the DWG, CDC, and other partners developed the MS Excel-based Pre-Campaign Dashboard (PCDB) [9]. Local government areas -level maps are embedded in the PCDB to automatically indicate, by ward, completed pre-campaign activity, with dynamic filtering for four preparedness review time points prior to the planned SIA start date (3 weeks, 2 weeks, 1 week, and 3 days) (Table 1). The LGA-level PCDB has 14 main indicators comprised of 36 sub-components. Pre-campaign dashboard data are compiled at the state level and reported to the NEOC by each of the preparedness review time points. The PCDB has 19 indicators to assess overall state-level preparedness (Figure 1).

**Table 1:** pre-campaign dashboard indicators to monitor the level of preparedness for supplementary immunization activities in implementing States, Nigeria, 2012-2020

Phases	Indicators
Indicators due as at 3 weeks to implementation	Implementation of planned social mobilization activities, high-risk operational plan, local government area (LGA)-level task force meeting, and meeting of the ward selection committee.
Indicators due as at 2 weeks to implementation	Updated micro plan, training conducted at the LGA level, social mobilization fund received at the LGA level, and counterpart fund.
Indicators due as at 1 week to implementation	Border synchronization planning meeting and transport logistic funds distribution plan.
Indicators due as at 3 days to implementation	Security assessment, transport logistic fund received, required vaccine availability at LGA level, and required funds for fueling and other miscellaneous activities available.

**During supplementary immunization activities:** for each polio SIA during 2016-2020, administrative data on the number of children reported as immunized and vaccine doses used were collected by vaccination teams using a standard paper form. Data were aggregated by ward focal persons and reported to LGAs daily where they were entered using a WHO Nigeria-designed Microsoft Access database tool and submitted to the state. Each state submitted merged data to the WHO zonal office. Upon receiving the merged data from zones, the DWG conducted additional data quality checks, provided feedback, and merged data from all implementing states. Intra-campaign data were summarized on the total number of children immunized, doses used, and wastage rate by administrative level (Figure 1).

**After supplementary immunization activities:** After the 4-day SIAs, mop-up activities in under-performing areas were conducted on the fifth day. After this, independent monitors survey households and camps to assess the SIA vaccination status of children. In each implementing LGA, end-process independent monitoring (IM) data were collected for eight randomly selected wards. In each, three settlements were selected, and 15 households were randomly sampled per settlement, totaling 360 households per LGA [9]. For outside-the-household monitoring (e.g. at markets and transit points), caretakers of 240 children were sampled [10]. The DWG analyzed the percentage of missed children, the reasons for being missed, and the source of information about the SIA. The Lot Quality Assurance Sampling (LQAS) is the method used to assess the polio SIA quality and the LQAS surveys were conducted in purposively sampled LGAs [11]. Data were collected using an Open Data Kit online checklist by independent, trained surveyors (different than those above) starting after the mop-up day. Settlements were selected by the DWG for each sampled LGA. Supplementary immunization activities in LGAs were categorized as “passed” at ≥90% threshold or “failed” at <90% (>3 missed children out of 60 sampled children) [11,12]. The DWG provided comprehensive post-campaign analyses two weeks after each SIA to the NEOC, including the list of poor-performing sampled settlements in LGAs that failed LQAS (Figure 1).

**Poliovirus surveillance:** acute flaccid paralysis (AFP) surveillance data are collected from Health centers; LGA Disease Surveillance and Notification Officers collect AFP surveillance and investigation data. These paper forms are entered and collated in the same manner as SIA data. The DWG compiles data weekly from AFP and environmental surveillance (sewage sampling) and results of laboratory testing, including spatial analyses and caretaker recall dose histories for children with non-polio AFP.

## Results

A total of 13 datasets were generated during each oral poliovirus vaccine supplementary immunization activity.

**Figure 2:**
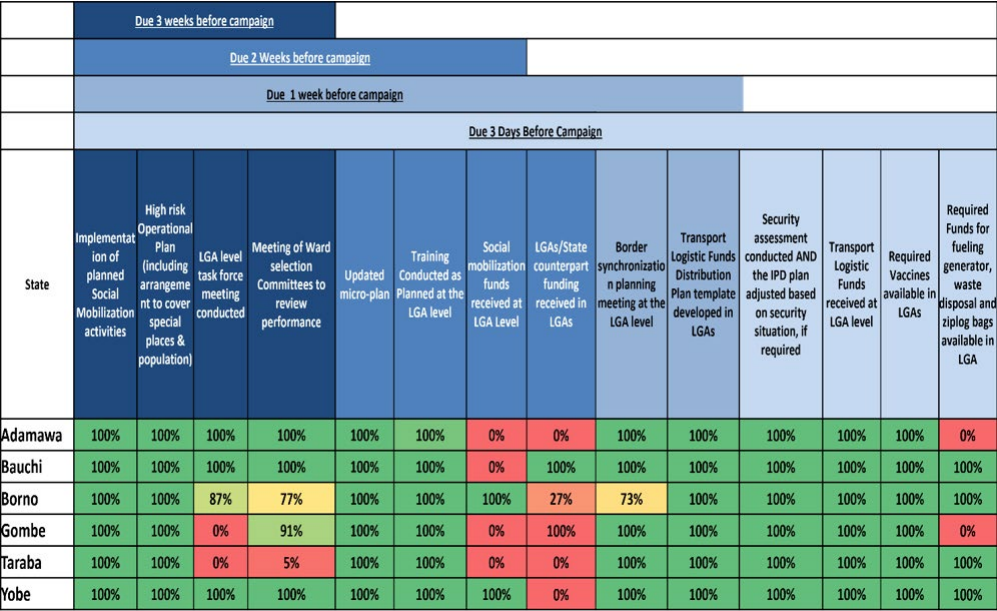
local government area level pre-campaign dashboard for selected States at 3 days prior to scheduled implementation, February 2020 National immunization days

**Before campaign:** for an NID, the PCDB tool aggregated and summarized 27,864 data points, i.e. 36 sub-indicators for each of 774 LGAs, into a dashboard graphic for 14 main indicators which shows the proportion of participating LGAs in each state that have conducted all preparation activities by the due timeline. For the polio SIAs during August 2016-February 2020, the level of preparedness 3 days before the start of OPV polio campaigns on average was 87% with the lowest at 77% and the highest at 95%. A representable PCDB for the February 2020 NIDs is indicated in Figure 2. Every implementing state was monitored based on the main 14 LGA level PCDB indicators by milestone review due time. National emergency operation centre review at each of the four-time points was instrumental in identifying delays in pending pre-campaign activities in time to deploy support if needed. The overall levels of preparedness gradually improved due to the follow-up made after sharing the PCDB information for each phase. In the 17 southern states, the EOC Strategy Work Group rescheduled campaigns in order to provide more time for improved preparedness.

**During and after the campaign:** during the 4 days of the campaign period, tally sheet data quantifying the number of children immunized, and vaccine doses used were collected and analyzed. Following the completion of the SIA, IM and LQAS surveys were analyzed. Based on identified gaps, NEOC has taken corrective actions on upcoming SIAs. On average, 53.3 million children aged <5 years were reported as immunized per NID round by administrative data. Wastage rates declined due to the feedback given to each state and corrective actions taken: the maximum wastage rate was 17% in 2016, and the minimum wastage rate was 5% more recently. Although biased to undercount, the average percentage of reported missed children by IM was 2%, with a maximum of 3% in 2018. Information on reasons for missing children and sources of information were used to develop communication strategies. The LQAS data were used to assess the quality of the SIA and identify low-performing areas: LGAs that failed LQAS conducted revaccination in poor-performing settlements. Based on the trend analysis, 20-25% of sampled LGAs have failed in LQAS with marked declines over time. The highest LQAS result was achieved in April 2019 NIDs, with 88% of LGAs passing at ≥90%. There was no NIDs in 2022 due to the COVID-19 pandemic and movement restriction.

**Non-polio acute flaccid paralysis dose histories:** caretaker dose history recall indicated >95% of children with non-polio AFP had >3 doses of OPV from SIA and routine immunization. In 2018 and 2019, 1% of the children had received no OPV and this triggered investigations to determine fundamental reasons and aided the identification of high-risk LGAs for routine immunization intensification.

## Discussion

We have demonstrated that the provision of complete, timely, and quality surveillance and SIA information contributed to evidence-based decision-making in the polio eradication program in Nigeria. The PCDB provided the ability to process data in a timely and logical manner to generate evidence-based information. This helped the NEOC to make informed decisions based on the assessment of each state’s level of preparedness to implement each OPV SIA. The PCDB information triggered teleconferences with low-performing states. Based on PCDB information, NEOC dispatched a team to implement states to support preparedness. As a result, the pre-campaign dashboard was a key essential information tool to monitor and improve preparedness at the state and LGA levels, thereby contributing to the overall success of the campaigns.

The LQAS analysis feedback contributed to improved revaccination efforts in poor-performing LGAs. Based on historical information, the sub-optimal LQAS results in the southern part of the country were attributed to low preparedness and poor team performance during implementation. This indicates that the data analysis showed areas that are performing well, highlighted places in which coverage has generally been low using spatial analysis, and provided reasons for such low coverage.

The weekly surveillance update shared with all polio partners in the Nigerian polio program provided information on WPV1, circulating vaccine-derived poliovirus type-2 (cVDPV2), compatible cases, vaccination status of AFP cases, non-reporting LGAs, and other key surveillance indicators such as non-polio enterovirus (NPENT) isolation rate in AFP stool samples and Sabin-like poliovirus rate due to vaccine strains. The information provided has enabled program managers to make evidence-based decisions, including mitigation plans in poor-performing LGAs. Surveillance and immunization information has become an important input for rational decision-making and improving surveillance and immunization performance for the Nigeria polio program.

While we have shown the importance of information for decision-making processes, its use for decision-making has its own limitations. First, late and incomplete data reporting was the major challenge in analyzing data collected during OPV SIAs. More specifically, the vaccine administrative data for the SIA was a challenge and provided either very high or negative wastage rates. Secondly, getting a timely response from LGAs for data quality issues identified at the national level was sub-optimal [13]. Despite these limitations, we observed that the use of information has contributed to evidence-based decision-making in Nigeria as the DWG made efforts to review and improve the various data instruments over time, and training sessions were conducted to build the capacity of the state and LGA level data officers on data quality improvement needs.

## Conclusion

The provision of quality and timely data to generate polio surveillance and SIA information contributed to evidence-based decision-making in the polio eradication program in Nigeria. In a similar report from Kenya, information management played a valuable role in enhancing the quality and use of data for evidence-based planning and decision-making [11]. The PCDB information triggered teleconferences with slowly progressing states. Low PCDB preparedness scores by 1 week and/or 3 days prior to the scheduled SIA date prompted the NEOC to put some SIAs on hold in the States because SIA quality would be at risk. Based on the trends in LQAS results during 2016-2020, SIA quality improved in initially poorly performing LGAs aided by DWG spatial analyses. Incorporating data and evidence-based information for the assessment of the Nigerian polio program activities and the formulation of improvement objectives targeted to address specific issues contributed to more resilient and effective polio eradication efforts, especially with the setback of WPV cases resurgence in 2016 after going two years without any case. The shared approach of data collection and reporting, multiple channels of data verification, and the multi-organization structure of the DWG at the NEOC enabled a process of collaboration, partnership, transparency, and accountability on the provision of quality and timely data in the Nigerian polio program.

**Disclaimer:** the findings and conclusions in this report are those of the authors and do not necessarily represent the official position of the U.S. Centers for Disease Control and Prevention.
